# Incidence, clinical implications and impact on public health of infections with *Shigella spp*. and entero-invasive *Escherichia coli* (EIEC): results of a multicenter cross-sectional study in the Netherlands during 2016–2017

**DOI:** 10.1186/s12879-019-4659-y

**Published:** 2019-12-09

**Authors:** Maaike J. C. van den Beld, Esther Warmelink, Alexander W. Friedrich, Frans A. G. Reubsaet, Maarten Schipper, Richard F. de Boer, Daan W. Notermans, Mariska W. F. Petrignani, Evert van Zanten, John W. A. Rossen, Ingrid H. M. Friesema, A. M. D. ( Mirjam) Kooistra-Smid, on behalf of the IBESS working group, A. P. van Dam, S. Svraka-Latifovic, A. M. D. Kooistra-Smid, J. J. Verweij, L. E. S. Bruijnesteijn van Coppenraet, K. Waar, M. Hermans, D. L. J. Hess, L. J. M. van Mook, A. M. C. Bergmans, R. R. Jansen, J. H. B. van de Bovenkamp, A. A. Demeulemeester, E. Reinders, C. F. M. Linssen

**Affiliations:** 1 Infectious Disease Research, Diagnostics and laboratory Surveillance, Centre for Infectious Disease Control, National Institute for Public Health and the Environment, Bilthoven, The Netherlands; 2 Department of Medical Microbiology and Infection Prevention, University of Groningen, University Medical Center Groningen, Groningen, the Netherlands; 3 Public health service GGD Groningen, Groningen, the Netherlands; 4 Department of Statistics, Informatics and Mathematical Modeling, National Institute for Public Health and the Environment (RIVM), Bilthoven, the Netherlands; 5 Certe, Department of Medical Microbiology, Groningen, the Netherlands; 6 Public health service GGD Amsterdam, Amsterdam, the Netherlands; 7 National Coordination Centre for Communicable Disease Control, Centre for Infectious Disease Control, National Institute for Public Health and the Environment, Bilthoven, The Netherlands; 8 Infectious Diseases, Epidemiology and Surveillance, Centre for Infectious Disease Control, National Institute for Public Health and the Environment, Bilthoven, the Netherlands

**Keywords:** *Shigella*, Shigellosis, Entero-invasive *Escherichia coli*, EIEC, Clinical implications, Public health, Incidence, Infectious disease control, Guidelines, Case definition

## Abstract

**Background:**

*Shigella spp.* and entero-invasive *E. coli* (EIEC) use the same invasive mechanism to cause diarrheal diseases. Public health regulations apply only to *Shigella spp*. infections, but are hampered by the lack of simple methods to distinguish them from EIEC. In the last decades, molecular methods for detecting *Shigella spp*. and EIEC were implemented in medical microbiological laboratories (MMLs)**.** However, shigellosis cases identified with molecular techniques alone are not notifiable in most countries. Our study investigates the impact of EIEC versus *Shigella spp.* infections and molecular diagnosed shigellosis versus culture confirmed shigellosis for re-examination of the rationale for the current public health regulations.

**Methods:**

In this multicenter cross-sectional study, fecal samples of patients suspected for gastro-enteritis, referred to 15 MMLs in the Netherlands, were screened by PCR for *Shigella spp.* or EIEC. Samples were cultured to discriminate between the two pathogens. We compared risk factors, symptoms, severity of disease, secondary infections and socio-economic consequences for (i) culture-confirmed *Shigella spp.* versus culture-confirmed EIEC cases (ii) culture positive versus PCR positive only shigellosis cases.

**Results:**

In 2016–2017, 777 PCR positive fecal samples with patient data were included, 254 of these were culture-confirmed shigellosis cases and 32 were culture-confirmed EIEC cases. EIEC cases were more likely to report ingestion of contaminated food and were less likely to be men who have sex with men (MSM). Both pathogens were shown to cause serious disease although differences in specific symptoms were observed. Culture-negative but PCR positive cases were more likely report travel or ingestion of contaminated food and were less likely to be MSM than culture-positive cases. Culture-negative cases were more likely to suffer from multiple symptoms. No differences in degree of secondary infections were observed between *Shigella spp.* and EIEC, and culture-negative and culture-positive cases.

**Conclusions:**

No convincing evidence was found to support the current guidelines that employs different measures based on species or detection method. Therefore, culture and molecular detection methods for *Shigella spp*. and EIEC should be considered equivalent for case definition and public health regulations regarding shigellosis. Differences were found regarding risks factors, indicating that different prevention strategies may be required.

## Background

*Shigella spp.* are one of the leading causes for diarrheal mortality and morbidity, predominantly in resource-restricted areas [1]. In resource-rich areas imported and domestically acquired shigellosis poses a substantial burden on public health due to the use of healthcare facilities, requirement for disease control measures, and a high number of disability adjusted life years [1–4].

Entero-invasive *Escherichia coli* (EIEC) is a pathotype of *E. coli* that causes diarrhea, using the same invasive mechanisms as *Shigella spp.* [5, 6]. *Shigella spp*. and EIEC result from the convergent evolution of ancestral *E. coli* which independently acquired the large invasion virulence plasmid (pINV) on multiple occasions [7]. Genetically, *Shigella spp.* and EIEC share virulence genes. Furthermore, they are related to such an extent that they should be classified as one species together with other *E. coli* pathotypes and commensals, however the current designation of two genera is maintained [8, 9].

Molecular detection of *Shigella spp.* and EIEC from fecal samples based on the presence of virulence genes such as the *ipaH*-gene greatly improved diagnostics [10]. However, because of their shared characteristics, differentiating EIEC from S*higella spp*. in the routine medical microbiology laboratory (MML) is difficult. In the last decade, multiple research groups developed molecular markers or methods that aimed to distinguish *Shigella spp*. from EIEC [8, 11–15]. While most of these methods were able to correctly identify isolates in the isolate set of their developers, it was demonstrated that these molecular methods have difficulties identifying other isolates, particularly EIEC isolates ( [8, 13, 14] van den Beld et al., submitted). These complications with molecular assay development are probably caused by the high heterogeneity of EIEC isolates, leading to the identification of subgroups rather than the whole pathotype EIEC ( [8, 16], van den Beld et al., submitted). Moreover, differentiating cultured isolates based on physiological and biochemical properties is complicated, as EIEC can display either an *E. coli*-like profile or a more inactive Shigella-like profile, and all profiles in-between [17]. Nevertheless, culturing is performed for several reasons. First, culturing for antimicrobial susceptibility testing is pivotal, as *Shigella spp*. are on the global priority list for antibiotic-resistant bacteria [18]. Second, to distinguish *Shigella spp*. from EIEC, culture-dependent identification methods are required, as molecular methods cannot be used for this purpose [5, 17]. This distinction is only important because EIEC infections are not notifiable in most countries while shigellosis is. Furthermore, in the Netherlands, as in many other countries, confirmed case definitions for shigellosis in control regulations specifically require the isolation of *Shigella spp.* [19–22].

Despite these advantages of culturing, culture methods for *Shigella spp*. are known to have limited sensitivity [23, 24]. Isolation of EIEC from fecal samples is even more challenging, as selective agar plates are based on biochemical properties such as fermentation of lactose and decarboxylation of lysine that EIEC shares with some other *Enterobacteriaceae* present in the gut [25].

The similarity between *Shigella spp.* and EIEC makes regulations, which require the notification of *Shigella* spp. but not EIEC, difficult to apply in practice by both laboratories and physicians. Apart from some studies that describe the infectious potential of EIEC and their ability to cause food related outbreaks, limited research has been performed on this subject [26–30]. Therefore, little is known of the severity and sequela of EIEC with respect to the incidence and impact on individual patients or public health.

Additionally, public health authorities struggle with the control of shigellosis cases identified with molecular techniques alone, because the impact of these cases for patients and public health is also not well defined [31, 32]. Two studies have looked at differences in case demographics, risk factors, and disease outcomes of shigellosis in culture-positive cases versus culture-negative cases [31, 33]. However, in both studies the proportion of EIEC infections amongst the culture-negative cases was unknown, and in one of the studies the data was biased because laboratory testing was unevenly distributed between laboratories that used either culture methods or molecular methods [31, 33].

To obtain a more complete insight into the implications of infections with *Shigella spp.* and EIEC and the challenges regarding their detection, distinction and control measures, a multicenter cross-sectional observational study was performed in the Netherlands ‘the Invasive Bacteria *E. coli-Shigella* Study’ (IBESS). We compared results with regard to incidence, clinical implications and impact on public health for (i), infections with EIEC or *Shigella spp.* and (ii), culture confirmed shigellosis versus molecular detected shigellosis. With this study, more evidence is obtained for improvements of the guidelines for control of shigellosis.

## Methods

### Study design and inclusion criteria

**Fig. 1:**
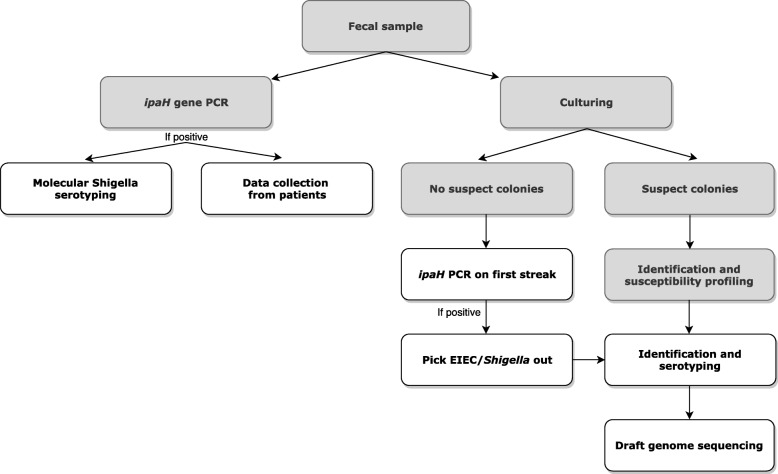
Overview of the design of the IBESS-study. Grey boxes = activities performed by participating laboratories. White boxes = activities performed by the IBESS-study group

During 2016 and 2017, 15 medical microbiological laboratories (MMLs) and their respective public health services (PHS) participated in this study. Fecal samples from patients suspected for gastro-enteritis that were referred to one of the participating MMLs for regular diagnostics, in which *Shigella spp.* or EIEC was detected with molecular methods, were included. After inclusion, the DNA eluate of the fecal sample and, if available, a cultured isolate were sent to the study group. A molecular algorithm based on the ratio of Ct-values of the *ipaH* gene and the *Shigella wzx* genes was used to serotype directly from fecal samples [34]. In addition, all obtained isolates were identified and serotyped with classical methods as previously described [34] (Fig. 1). Furthermore, clinical and epidemiological data were collected from all included patients (Fig. 1).

### Data collection

Data was collected from patients using two approaches. For fecal samples of which a *Shigella spp.* was isolated, which are notifiable under current regulations, PHS performed source tracing according to the guidelines. Patients were informed about the study and requested to give consent for their participation in this study after completion of the regular survey regarding source tracing for shigellosis. After consent, an infectious disease nurse from the study group contacted them by telephone. They were informed again about the study and, after their further consent, subjected to a single survey to collect additional clinical and epidemiological data. In contrast, for fecal samples of which *Shigella spp.* or EIEC was detected with molecular methods only or from which an EIEC isolate was cultured, an infectious disease nurse from the study group contacted the physicians of the patients first to request their permission for contacting the patients. After their consent, the patient was contacted by the infectious disease nurse for collection of data as described above.

### Incidence

Incidence of *Shigella spp.* for the years 2016 and 2017 was calculated using the numbers of national shigellosis notifications as numerator and residents in the Netherlands on 1 January 2017 as denominator. A multiplier of 53 was applied, as for one notified shigellosis case, 53 cases have been estimated to be to be missed in the Netherlands due to under-reporting and under-diagnosing [35].

The proportion of *Shigella spp.* isolates included in this study from total notified shigellosis cases was determined, and was used to calculate the incidence of EIEC by extrapolating the proportion to the EIEC isolates included in this study to a national level. However, the multiplier that was modelled to calculate the community incidence for shigellosis is not suitable to use for EIEC cases. In the algorithm of Haagsma et al., the sensitivity of the laboratory analysis and the percentage of bloody diarrhea are important factors used to correct for under-reporting and under-diagnosing [35]. However, these factors are known to vary among different enteric pathogens. From an earlier study, it is known that only 5 out of 16 MMLs performed culture of EIEC in the Netherlands. This proportion was multiplied with the laboratory analysis sensitivity of 0.63 as proposed for shigellosis by Haagsma et al., resulting in a sensitivity of 0.20 for laboratory analysis of EIEC [35]. This factor was used in the calculation of a specific multiplier for the community incidence of EIEC infections, together with the fraction of patients with EIEC infections that reported bloody diarrhea in the study described here, which was 0.16. The country specific parameters for the Netherlands as reported earlier were maintained [35].

### Data and analysis

The following patient variables were collected: risk factors for infection, clinical symptoms, presence and number of related patients indicative for the degree of secondary infections, and socio-economic consequences. The patients themselves provided variables in a telephone interview. In an effort to assess the degree of secondary infections, they were specifically asked if they knew of other people who fell ill before or after their own onset of symptoms to exclude common sources of infection. All reported underlying diseases and medication use reported by patients were stratified into categories and considered as factors. Clinical symptoms were self-reported and not measured or verified by a physician. To assess the severity of the disease for individual patients, the total number of reported symptoms by each individual patient was added up. Additionally, two severity scales, the de Wit scale and the modified Vesikari-scale (MVS), were applied in which higher scores indicated more severe course of disease [36, 37]. Co-infections with other enteric pathogens were reported by the participating MMLs if detected by molecular methods, culture or microscopy. The study group determined identity of the obtained isolates and bacterial load in fecal samples was estimated by cycle-threshold (Ct) values of the *ipaH* gene following from the molecular algorithm that was used for the direct *Shigella* serotyping in fecal samples. Bacterial load and species designations were only considered in the comparison of culture-positive to culture-negative shigellosis cases because it is known that culture rates increase with an increase in bacterial load (decrease in Ct-value) and that *S. sonnei* is easier to detect by culture than *S. flexneri* [23, 38].

As data was actively retrieved, missing values were scarce, and included as missing in the statistical analysis. Comparisons were made for patients with *Shigella spp.* to patients with EIEC to assess support for the current guidelines in which culture confirmed infections with *Shigella spp.* are notifiable, while infections with EIEC are not. Additionally, culture-positive cases were compared with culture-negative shigellosis cases, to assess support for the current case definition of shigellosis, in which only culture-confirmed cases are notifiable. To examine if large dissimilarities exist for infections with different *Shigella spp*., infections with cultured *S. flexneri* and *S. sonnei* were also compared.

For the comparison of culture-positive with culture-negative cases, only infections with *S. flexneri* and *S. sonnei* were analyzed. S*. boydii* and *S. dysenteriae* were excluded because of low case numbers (*n* < 5). Molecular *Shigella* serotyping by real-time PCR in culture-negative samples was based on the *ipaH* gene, the *S. sonnei wzx* gene, and the *S. flexneri wzx*_*1–5*_ or *wzx*_*6*_ gene as described before [34]. As the *ipaH* gene is present in multiple copies, in contrast to the *wzx* genes, their Ct-values should represent these ratios to confirm the direct identification of *S. sonnei* and *S. flexneri* by molecular methods. Infections were defined as culture-positive if *S. flexneri* or *S. sonnei* was isolated from the fecal sample.

Differences in risks factors between groups were calculated with univariate and multivariate analyses using logistic regression. All variables with *p* < 0.20 in the univariate analysis were included in the multivariate model, where the least significant variables were one-by-one eliminated until all remaining variables reached significance (*p* value< 0.05). Analyses were performed using SAS® software version 9.4 (SAS Institute Inc., Cary, NC, USA), odds ratios with their 95% confidence intervals were calculated using the beta and standard error (SE) values from the logistic regression models.

Differences in symptoms, severity of disease, degree of secondary infections and socio-economic consequences were calculated using multivariate analyses with the following confounders: sex, age, MSM contact, co-infections, effect of underlying diseases or medication use, and Ct values as measure for bacterial load. In the multivariate analyses for the comparison of culture-positive infections and culture-negative infections, the confounder “species” was added, because *S. flexneri* showed lower culture rates (38%) than *S. sonnei* (63%). These analyses were performed using R. version 3.4.3 [39]. and significance was defined as *p* < 0.05.

## Results

**Fig. 2:**
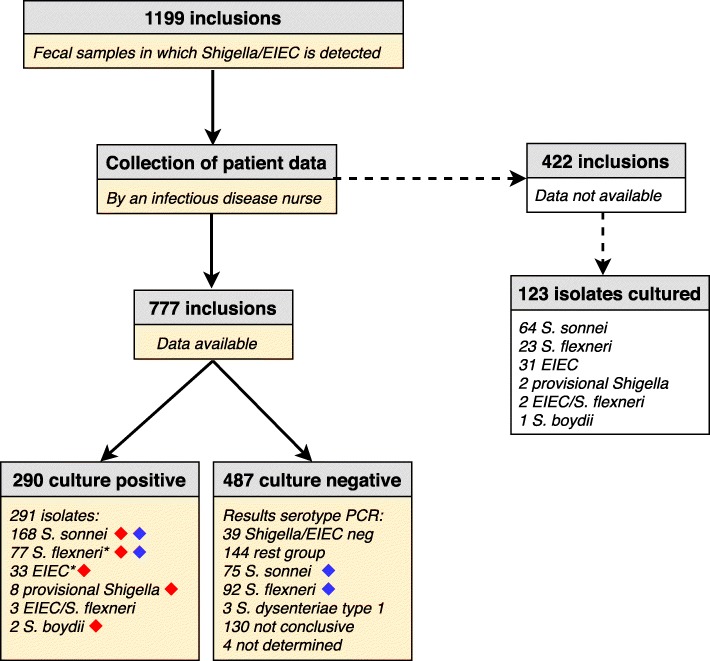
Flowchart of inclusions in the study. Yellow boxes = data used in this study. White boxes = data not used in this study. Red diamonds = Data of patients from whom these isolates were obtained were used in the comparison of *Shigella spp.* with EIEC. Blue diamonds = Data of patients from whom an *S. sonnei* or *S. flexneri* isolate was obtained or detected in the fecal samples were used in the comparison of culture-positive cases with culture-negative cases. ^*^one *S. flexneri* and one EIEC isolate were excluded from analysis, because they caused a double-infection

In our study, 1199 PCR positive fecal samples were included over the course of 2 years (Fig. 2). From the fecal samples, 414 isolates were cultured and initially identified as 232 *S. sonnei*, 100 *S. flexneri*, 64 EIEC, 10 provisional *Shigella*, 3 *S. boydii*, for the remaining 5 isolates a distinction between *S. flexneri* and EIEC could not be made. *Shigella* were called provisional if the serotype could not be determined, or if the established serotype did not match with the phenotype. In total, 777 (65%) patients provided clinical and epidemiological data. Samples of these patients were included for the comparisons described below (Fig. 2). In total, 290 of the 777 patients had a culture-positive infection. The data of patients from whom a *S. sonnei*, *S. flexneri*, *S. boydii* or provisional *Shigella* (*n* = 255) isolate was obtained were used in the comparison to patients of whom an EIEC isolate (*n* = 33) was cultured (Fig. 2). For comparison of culture positive cases to culture negative cases, only data from patients of which *S. sonnei* or *S. flexneri* was cultured (*n* = 245) were compared to patients of which *S. sonnei* or *S. flexneri* was molecularly detected (*n* = 167) (Fig. 2). One *S. flexneri* and one EIEC isolate were excluded from all analyses because they were cultured from the same fecal sample.

Assessment of the sensitivity and specificity of the molecular *S. flexneri* and *S. sonnei* serotyping directly from fecal samples resulted in a sensitivity of 77 and 75%, and a specificity of 98 and 99% respectively.

### Incidence

During 2016 and 2017, 873 cases of shigellosis were notified to the health authorities, resulting in an average of 436.5 cases each year. The total number of residents in the Netherlands on 1 January 2017 was 17,081,507, resulting in an estimated incidence 135 shigellosis cases per 100,000 residents per year in the Netherlands during 2016 and 2017. Almost 40 % (39.5%) of all notified shigellosis cases were included in this study. We assumed the same ratio of EIEC cases having been included in our study and multiplied their number by 2.53 to estimate the national EIEC incidence rates. As 64 EIEC isolates were cultured, this resulted in 160 EIEC cases in 2 years, i.e., 80 per year. From the estimation for specific EIEC community incidence followed that a multiplier of 265 should be applied, see Additional file 1 for calculations. This resulted in 80*265 = 21,200 cases in the Dutch population, translated to a community incidence for EIEC of 124 cases per 100,000 residents per year in the Netherlands during 2016 and 2017.

### Risks factors

Our results showed that patients with EIEC infections were more likely to report ingestion of suspected contaminated food or water (OR: 3.04 (1.44–6.42)) and less likely to report MSM contact (OR: 0.21 (0.05–0.98)) as source for infection compared to patients with *Shigella spp.* (Table 1).

**Table 1:** Risk factors of infections with EIEC and *Shigella*, and culture-positive and culture-negative shigellosis

Risk factors	EIEC^a, b^ (*n* = 32)	*Shigella spp.*^a^ (*n* = 254)	Univariate OR (95% CI)	Multivariate OR (95% CI)	Culture +/ PCR + ^a, b^ (*n* = 244)	Culture - / PCR + (*n* = 167)	Univariate OR (95% CI)	Multivariate OR (95% CI)
Sex of patient (female)	44%	46%	0.91 (0.43–1.91)		46%	53%	0.76 (0.50–1.16)	
Age of patient (mean ± sd)	36.0 ± 20.4	38.9 ± 18.5	0.99 (0.97–1.01)		38.7 ± 18.8	41.1 ± 19.3	0.99 (0.98–1.00)	
Living in multi-person household	78%	74%	1.37 (0.57–3.33)		75%	80%	0.89 (0.58–1.35)	
Co-infection with other enteric pathogen	28%	13%	**2.72 (1.15–6.38)**		12%	11%	1.04 (0.54–1.99)	
Bacterial load (Ct-value, mean ± sd)					22.9 ± 4.6	25.3 ± 4.8	**0.90 (0.86–0.94)**	**0.88 (0.84–0.93)**
Species (*S. flexneri*)					31%	55%	**0.36 (0.23–0.55)**	**0.32 (0.19–0.54)**
Effect underlying disease/use of medication								
Higher infection risk	3%	20%	**0.18 (0.03–0.91)**		21%	17%	1.31 (0.82–2.08)	
More severe course	13%	7%	1.90 (0.69–5.20)		7%	6%	1.28 (0.65–2.55)	
Higher infection risk + more severe course	9%	10%	1.04 (0.35–3.07)		9%	11%	0.82 (0.46–1.46)	
Unknown effect	13%	6%	2.25 (0.81–6.24)		7%	11%	0.71 (0.39–1.31)	
Travel history	88%	60%	**4.62 (1.57–13.57)**		57%	83%	**0.26 (0.16–0.43)**	**0.40 (0.20–0.78)**
Regions:								
South America	13%	4%	**3.07 (1.04–9.04)**		3%	5%	0.65 (0.25–1.69)	
Central America	13%	6%	1.73 (0.62–4.79)		5%	5%	0.95 (0.41–2.19)	
Asia	34%	17%	1.77 (0.85–3.67)		12%	26%	**0.45 (0.26–0.77)**	
Africa	25%	28%	0.79 (0.36–1.71)		30%	44%	0.65 (0.41–1.01)	
Europe	3%	6%	0.49 (0.09–2.78)		5%	2%	2.53 (0.84–7.68)	
Source of infection (suspected by patient):								
Contaminated food/water	53%	26%	**3.04 (1.44–6.42)**	**3.04 (1.44–6.42)**	27%	64%	**0.33 (0.23–0.48)**	**0.38 (0.24–0.61)**
MSM contact	3%	22%	**0.21 (0.05–0.98)**	**0.21 (0.05–0.98)**	24%	7%	**2.84 (1.65–4.90)**	**3.22 (1.70–6.09)**
Unknown	38%	45%	1.25 (0.58–2.71)	1.25 (0.58–2.71)	42%	20%	**1.70 (1.14–2.54)**	**1.85 (1.17–2.92)**
Infection occupation related	9%	4%	1.64 (0.83–3.25)		3%	8%	0.62 (0.38–1.03)	

*OR* Odds ratio, *CI* 95% confidence interval, *sd* Standard deviation. ^a^one *S. flexneri* and one EIEC isolate were excluded from analysis, because they caused a double-infection. ^b^EIEC and culture + /PCR + were considered as cases, *Shigella spp.* and culture −/ PCR + as controls. Bold values indicate significant results with *p*-values <0.05.

As expected, Ct-values were approximately three Ct lower for the culture-positive shigellosis cases (OR: 0.88 (0.84–0.93)) than for culture-negative cases. Additionally, the proportion of *S. flexneri* in culture-positive infections was lower than the proportion in culture-negative infections (OR: 0.32 (0.19–0.54)). Furthermore, assessment of risk factors revealed that culture-positive cases travelled less (OR: 0.40 (0.20–0.78)) and were more likely to report MSM contact (OR: 3.22 (1.70–6.09)) or an unknown infection source (OR: 1.85 (1.17–2.92)) than culture-negative cases. In addition, culture-positive cases were less likely to report ingestion of suspected contaminated food or water as infection source than culture-negative cases (OR: 0.38 (0.24–0.61)) (Table 1).

### Symptoms, severity of disease and socio-economic consequences

Patients with EIEC infections reported suffering for longer from diarrhea than patients with *Shigella spp.* infection. In addition, the maximum vomiting frequency was higher for patients with EIEC infections (Table 2). Although patients with EIEC were symptomatic longer, they exhibited fewer symptoms and scoring lower on the de Wit scale than patients with *Shigella spp.* In contrast, no significant difference in severity was calculated using the MVS scale (Table 2). For socio-economic consequences, patients with EIEC infections were more likely to visit a general practitioner (GP) and to have a shorter stay when hospitalized than patients with a *Shigella spp.* infection (Table 3).

**Table 2:** Symptoms and severity of infections with EIEC and *Shigella*, and culture-positive and culture-negative shigellosis

Symptoms and severity	EIEC^a, b^ (n = 32)	*Shigella spp.*^a^ (*n* = 254)	Univariate model, *p*-value	Multivariate model, *p*-value	Culture +/ PCR + ^a, b^ (*n* = 244)	Culture - / PCR + (*n* = 167)	Univariate model, *p*-value	Multivariate model, *p*-value
Blood in stool (% present)	16	39	**0.005**	0.051	39	38	0.901	0.679
Mucus in stool (% present)	47	58	0.222	0.290	58	54	0.508	0.688
Abdominal pain (% present)	59	74	0.082	0.108	75	71	0.330	0.945
Abdominal cramps (% present)	72	82	0.194	0.115	82	83	0.662	0.310
Nausea (% present)	56	44	0.209	0.568	45	54	0.066	**0.041**
Headache (% present)	22	33	0.187	0.052	32	40	0.108	0.086
Fever (% present)	47	60	0.164	0.248	59	56	0.582	0.420
When fever, duration in days (median (IQR))	3 (2.5–4.5)	2 (1–4)	0.334	0.165	2 (1–4)	2 (1–4)	0.802	0.698
When fever, maximum temperature (mean ± sd)	40.0 ± 0.7	39.4 ± 0.9	0.063	0.413	39.4 ± 0.9	39.2 ± 0.8	0.084	0.179
Diarrhea (% present)	97	97	0.907	0.776	98	99	0.349	0.303
When diarrhea, duration in days (median (IQR))	14 (7–19.5)	10 (6–14)	**<0.001**	**<0.001**	9.5 (6–14)	14 (8–24)	**<0.001**	**0.001**
When diarrhea, frequency in 24H (median (IQR))	8 (6–14)	9 (6–15)	0.855	0.796	10 (6–15)	10 (6–16)	0.486	0.185
Vomiting (% present)	28	28	0.979	0.809	29	37	0.073	**0.026**
When vomiting, duration in days (median (IQR))	2 (1–3)	1 (1–3)	0.508	0.929	1 (1–3)	2 (1–3)	**0.033**	0.167
When vomiting, frequency in 24H (median (IQR))	3 (2–8)	2 (1–4)	0.166	**0.001**	2 (1–4)	3 (1–5.8)	0.525	**0.027**
Total number of symptoms (median (IQR))	4 (3.0–5.3)	5 (4–6)	**0.006**	**0.006**	5 (4–6)	5 (4–6)	0.519	0.104
Severity scores:								
- de Wit et al. (mean ± sd)	6.4 ± 2.6	7.5 ± 2.7	**0.033**	**0.045**	7.5 ± 2.7	7.7 ± 2.7	0.380	0.132
- Modified vesikari (mean ± sd)	7.4 ± 3.3	7.3 ± 2.8	0.852	0.943	7.3 ± 2.8	7.9 ± 2.8	**0.028**	**0.007**

*Sd* Standard deviation, *IQR* Interquartile range. ^a^one *S. flexneri* and one EIEC isolate were excluded from analysis, because they caused a double-infection. Bold values indicate significant results with *p*-values <0.05.

**Table 3:** Socio-economic consequences of infections with EIEC and *Shigella*, and culture-positive and culture-negative shigellosis

Consequences	EIEC^a, b^ (*n* = 32)	*Shigella spp.*^a^ (*n* = 254)	Univariate model, *p*-value	Multivariate model, *p*-value	Culture +/ PCR + ^a, b^ (*n* = 244)	Culture - / PCR + (*n* = 167)	Univariate model, *p*-value	Multivariate model, *p*-value
Bedrest (% present)	88	81	0.357	0.186	82	79	0.528	0.514
Leave of absence (% present)	56	53	0.709	0.703	53	47	0.220	0.737
When absence patient, duration in days (median (IQR))	5 (3.0–7.8)	4 (3–7)	0.882	0.401	4 (3–7)	7 (3–10)	**0.038**	**0.005**
When absence caretaker, duration in days (median (IQR))	0 (0–0)	0 (0–0)	0.554	0.185	0 (0–0)	0 (0–0)	0.389	0.171
Use of care facilities								
GP (% visited)	100	91	**0.015**	**0.037**	91	93	0.299	0.851
When GP visited, number of visits (median (IQR))	1.5 (1–2)	1 (1–2)	0.623	0.399	1 (1–2)	1 (1–2)	0.595	0.909
GP outside office hours (% visited)	9	9	0.989	0.537	9	10	0.694	0.757
Specialists (% visited)	16	13	0.732	0.830	13	16	0.388	0.965
When specialist visited, number of visits (median (IQR))	1 (1–2)	1 (1–1)	0.797	0.799	1 (1–1)	1 (1–2)	0.122	0.553
Emergency room (% visited)	9	10	0.933	0.781	10	5	0.072	0.074
Hospitalization (% hospitalized)	3	9	0.180	0.270	9	5	0.163	0.443
When hospitalized, duration in days (median (IQR))	1.5 (0.8–2.3)	3 (2–4)	0.179	**0.027**	3 (1.5–3.5)	3.5 (1–4.8)	0.244	0.648

*IQR* Interquartile range. ^a^one *S. flexneri* and one EIEC isolate were excluded from analysis, because they caused a double-infection. Bold values indicate significant results with *p*-values <0.05

Culture-negative cases were more likely to report nausea, longer duration of diarrhea, vomiting and higher frequencies of vomiting than culture-positive cases (Table 2). Moreover, the MVS score of culture-negative cases was significantly higher than that of culture-positive cases, while the de Wit scores showed no significant difference (Table 2). In addition, culture-negative cases were more likely to report longer absence from work compared to culture-positive cases (Table 3).

### Secondary infections

Because there was a lack of specific data about relationships between cases, the presence and number of self-reported related patients was used as a proxy for the degree of secondary infections. No significant differences in presence and number of self-reported related patients were found when comparing EIEC cases with shigellosis cases or when comparing culture-positive cases to culture-negative cases (Table 4).

**Table 4:** Degree secondary infections of infections with EIEC and *Shigella*, and culture-positive and culture-negative shigellosis

Secondary infections	EIEC^a, b^ (*n* = 32)	*Shigella spp.*^a^ (*n* = 254)	Univariate model, *p*-value	Multivariate model, *p*-value	Culture +/ PCR + ^a, b^ (*n* = 244)	Culture - / PCR + (*n* = 167)	Univariate model, *p*-value	Multivariate model, *p*-value
Related patients (% present)	47	39	0.393	0.785	40	39	0.865	0.930
When related patients, total number (median (IQR))	1 (1–2)	1 (1–2)	0.239	0.354	1 (1–2)	1 (1–3)	0.326	0.977

*IQR* Interquartile range. ^a^one *S. flexneri* and one EIEC isolate were excluded from analysis, because they caused a double-infection. Bold values indicate significant results with *p*-values <0.05

### Comparison of infections with cultured *S. flexneri* and *S. sonnei*

First, patients with *S. sonnei* were more likely to report (85%) abdominal cramps compared to *S. flexneri* (75%, *p* = 0.047). Second, no differences in total number of symptoms or disease severity were found. Third, patients with *S. sonnei* were more likely to self-report the presence of related patients (45%) than patients with *S. flexneri* (28%, *p* = 0.028), although the self-reported number of related patients did not differ. Fourth, for the socio-economic consequences, there were multiple differences: patients with *S. flexneri* were more likely to report longer absence from work (median 5 (3–9) days), multiple visits to their GP (average 2.1 visits), visits to specialists (21%) and hospitalization (17%) compared to patients with *S. sonnei* that reported a median of 4 (2–7), *p* = 0.001)) days of absence, an average of 1.6 GP visits (*p* = 0.049), 10% specialist visits (*p* = 0.015), and 5% hospitalization (*p* < 0.001).

## Discussion

This multicenter cross-sectional study was initiated to obtain more insight into the clinical implications and impact on public health of *Shigella spp.* and EIEC infections, by assessing differences in incidence, risk factors, symptoms, severity of disease, degree of secondary infections and the socio-economic consequences. Additionally, the clinical and public health relevance of detection of shigellosis with molecular methods only was investigated by comparing culture-positive shigellosis cases to culture-negative PCR positive shigellosis cases.

The comparison of infections with *Shigella spp.* and EIEC showed some differences, for which several hypotheses can be considered. Patients with EIEC infections were less likely to report MSM contact than patients with *Shigella spp.* Indeed, to our knowledge an EIEC outbreak among MSM has never been described. The higher infectious dose of EIEC could explain these lower transmission rates through the sexual route. Although, the claim of the higher infectious dose for EIEC is based on only one study from the 1970s, in which only two EIEC isolates were tested for pathogenicity at low dosages [28]. Despite the fact that patients with EIEC were symptomatic for longer periods, patients with *Shigella spp.* showed more symptoms simultaneously and a higher severity score on the de Wit scale. However, scores on the MVS scale were comparable. These discrepancies between the two disease severity scales were probably caused by the symptoms blood in stool and fever. Blood in stool and fever above 37.5 °C is double weighted in the de Wit scale, while in the MVS scale, blood in stool is not a factor and fever is double weighted only when temperature is above 38.4 °C. The differences regarding symptoms and disease provided no convincing evidence for a more severe course for one pathogen over the other. Patients with EIEC infections in our study were more likely to visit their GP. However, this is probably an artefact being a consequence of the healthcare system in the Netherlands, where only physicians can request laboratory confirmation, thus all patients diagnosed with an EIEC infection had visited their GP by definition. In contrast, PHS can also request laboratory confirmation of patients with shigellosis for cases that are identified during contact tracing [20]. This explains why not every patient with *Shigella spp*. visited their GP while patients diagnosed with EIEC did. Furthermore, similar percentages of patients infected with *Shigella spp*. and EIEC reported hospitalization, but patients infected with *Shigella spp.* were more likely to be admitted for longer periods. This may indicate a more severe disease course.

In our study, no biological evidence was found to support the current difference in approach for infections with *Shigella spp.* and EIEC, indicating that the disease control measures for EIEC should be the same as for *Shigella spp.* for several reasons. First, a reliable separation of these bacteria by MMLs is technically challenging and probably unachievable, as it is increasingly realized that they should be classified as one species as proposed by multiple research groups [8, 40]. Second, this study also associates EIEC infections with serious infections although minor differences in symptoms were observed compared to shigellosis. The pathogenic behavior of EIEC is also reflected in its involvement in multiple food-related outbreaks [26–30].

Although in some literature it is stated that *S. sonnei* causes milder forms of shigellosis than the other species of *Shigella* [41], in our study, as well as in other studies, no differences were found in disease severity when comparing *S. sonnei* and *S. flexneri* infections [42].

The limited sensitivity of culture from fecal samples should be further investigated. Causes for this phenomenon could be a low bacterial load, time between onset of symptoms and submission of the sample, and time between submission of the sample and diagnostic procedures. Nevertheless, the proportion of infections from which bacteria could be cultured in our study is comparable to other studies and is representative of the situation in the Netherlands and other areas [23, 43] (de Boer et al., manuscript in preparation).

Similar to others, we found that culture-negative cases were less likely to report MSM contact, more likely to report traveling and have a longer symptomatic period [31, 33]. Others explained that their culture negative cases reported higher travel rates because they are more likely to be infected by EIEC [33]. However, this explanation is not applicable to our study, as there is high certainty that EIEC infections were not included in our culture-negative group, because they were molecularly typed as *S. flexneri* or *S. sonnei* with a specificity of at least 98%. We suggest that laboratory confirmation might have been requested later in the course of the disease for travelers, reducing the chance of obtaining an isolate [23]. This is supported by the observation that the time between onset of disease and sample collection was longer for culture-negative cases in the earlier studies [31, 33]. Unfortunately, in our study, data about time of onset of disease was not available. In our study the total number of symptoms in culture-positive and culture-negative cases was comparable, in contrast to a previous study in which culture-negative cases were associated with a less severe course of disease [33] . However, culture-negative cases were more likely to suffer from nausea and vomiting and were symptomatic for longer than culture-positive cases. Moreover, culture-negative cases were associated with a longer absence from work, probably a consequence of their longer symptomatic period. Culture negative cases also had a higher score on the MVS scale, while the scores of de Wit scale were comparable. This discrepancy in the scales was probably caused by extended periods of diarrhea and higher frequency of vomiting in culture-negative cases; these factors are scored in the MVS scale but not in the de Wit scale. The results of the two severity scales are discordant throughout this study, indicating that interpretation of research into enteric infections depends highly on the severity scale chosen.

The current case definition for shigellosis was formulated when molecular methods were not implemented in routine diagnostics. Since their implementation, molecular methods have improved diagnostic capabilities, especially for organisms that are challenging to culture such as *Shigella spp.* and EIEC*.* However, because evidence about the meaning of PCR positive results was lacking, these methods are not yet incorporated into the case definition of shigellosis. Our study demonstrates that molecularly detected cases of shigellosis are comparable to culture confirmed shigellosis cases. There is no biological basis supporting the current case definition of shigellosis in which only culture confirmed cases are notifiable. Additionally, case control studies have demonstrated that the molecular detection of the *ipaH* gene in fecal samples was associated with cases rather than controls, and others showed that the sequence composition and quantity of *Shigella spp*. in culture-negative cases was comparable to culture-positive shigellosis cases [30, 38, 43–45]. Finally, guidelines from the European Union (EU), United States of America (USA) and Australia recently amended case definitions for shigellosis, and define molecular detected infections as probable cases, which in Australia should be notified, while in the EU and the USA individual countries or states should define their own notification criteria [19, 21, 22].

One of the strengths of this study is the inclusion of samples and patient data representative for the whole of the Netherlands, as a result of the collaboration with MMLs and PHS. A second strength is that the clinical outcomes and impact on public health of infections with EIEC were investigated; these have not often been described before [30]. A third strength is that the value of molecular detection of *Shigella spp.* versus culture was investigated in detail.

Limitations of this study are that the representation of species is based on the Dutch situation and therefore no *S. dysenteriae* isolates, and only a few *S. boydii* isolates were included in the comparison of outcomes of *Shigella spp.* and EIEC. Second, not all notified shigellosis cases were included, because not all laboratories in the Netherlands participated in the study, although participating laboratories had a reasonable national geographic distribution. Third, the study design introduces a bias towards more severe infections and certain demographics such as age and frailty, because only infections for which laboratory confirmation was requested were included. Fourth, no data was collected on date of onset of symptoms impeding correction for the comparison of symptomatic periods. Fifth, the number of self-reported related patients was used to estimate secondary infection rate. Although patients were asked to mention if they were aware of any other people that fell ill before or after them, common sources cannot be excluded with certainty using this method. Last, the clinical and epidemiological circumstances were not a result of objective measurements, but were dependent on the judgement and memory of the patients.

## Conclusions

This study provides evidence to reconsider incorporating molecular detection methods as well as infections with EIEC in the case definition and guidelines for disease control measures regarding shigellosis. As our study showed differences in risk factors between *Shigella spp*. infections and EIEC infections and between culture-positive and culture-negative shigellosis cases, the application of different prevention strategies deserves attention.

## Supplementary information

**Additional file 1.** Calculation of EIEC multiplier. In this file the calculation of the multipliers for the estimation of the Dutch community incidence for shigellosis and infections with EIEC were depicted, using equations as described by Haagsma et al. 2013.

## Data Availability

The data are not publicly available due to containment of information that could compromise research participants’ privacy.

## Abbreviations

Ct: Cycle threshold
DNA: Deoxyribonucleic acid
EIEC: Entero-invasive *Escherichia coli*
EU: European Union
GP: General practitioner
IQR: Inter-quartile range
METC: Medisch-ethische toetsing commissie (medical ethics review board)
MMLs: Medical microbiological laboratories
MSM: Men who have sex with men
MVS: Modified Vesikari-scale
OR: Odds ratio
PCR: Polymerase chain reaction
PHS: Public Health Services
*p*-value: probability-value
Spp.: Species

## References

[1] Diarrhoeal Diseases Collaborators GBD (2017). Estimates of global, regional, and national morbidity, mortality, and aetiologies of diarrhoeal diseases: a systematic analysis for the Global Burden of Disease Study 2015. Lancet Infect Dis.

[2] Khalil IA, Troeger C, Blacker BF, Rao PC, Brown A, Atherly DE (2018). Morbidity and mortality due to shigella and enterotoxigenic *Escherichia coli* diarrhoea: the Global Burden of Disease Study 1990-2016. Lancet Infect Dis.

[3] RIVM (2014). State of infectious Diseases in the Netherlands, 2013.

[4] Pijnacker R, Friesema IHM, Franz E, Van Pelt W (2017). Trends van shigellosemeldingen in Nederland, 1988-2015. Infectieziekten Bulletin.

[5] Lan R, Alles MC, Donohoe K, Martinez MB, Reeves PR (2004). Molecular evolutionary relationships of enteroinvasive *Escherichia coli* and *Shigella spp*. Infect Immun.

[6] Kaper JB, Nataro JP, Mobley HL (2004). Pathogenic *Escherichia coli*. Nat Rev Microbiol.

[7] Hale TL (1991). Genetic basis of virulence in *Shigella species*. Microbiol Rev.

[8] Pettengill EA, Pettengill JB, Binet R (2015). Phylogenetic analyses of *Shigella* and enteroinvasive *Escherichia coli* for the identification of molecular epidemiological markers: whole-genome comparative analysis does not support distinct genera designation. Front Microbiol.

[9] Brenner DJ, Family I, Krieg NR (1984). *Enterobacteriaceae* Rahn 1937, Nom. fam. cons. Opin. 15, Jud. Com. 1958, 73; Ewing, Farmer, and Brenner 1980, 674; Judicial Commission 1981, 104. Bergey’s Manual of Systematic Bacteriology.

[10] Hartman AB, Venkatesan M, Oaks EV, Buysse JM (1990). Sequence and molecular characterization of a multicopy invasion plasmid antigen gene, *ipaH*, of *Shigella flexneri*. J Bacteriol.

[11] Pavlovic M, Luze A, Konrad R, Berger A, Sing A, Busch U (2011). Development of a duplex real-time PCR for differentiation between E. coli and Shigella spp. J Appl Microbiol.

[12] Kim HJ, Ryu JO, Song JY, Kim HY (2017). Multiplex polymerase chain reaction for identification of Shigellae and four Shigella species using novel genetic markers screened by comparative genomics. Foodborne Pathog Dis.

[13] Chattaway MA, Schaefer U, Tewolde R, Dallman TJ, Jenkins C (2017). Identification of *Escherichia coli* and *Shigella Species* from whole-genome sequences. J Clin Microbiol.

[14] Dhakal R, Wang Q, Lan R, Howard P, Sintchenko V (2018). Novel multiplex PCR assay for identification and subtyping of enteroinvasive Escherichia coli and differentiation from Shigella based on target genes selected by comparative genomics. J Med Microbiol.

[15] Sahl JW, Morris CR, Emberger J, Fraser CM, Ochieng JB, Juma J (2015). Defining the phylogenomics of Shigella species: a pathway to diagnostics. J Clin Microbiol.

[16] Hazen TH, Leonard SR, Lampel KA, Lacher DW, Maurelli AT, Rasko DA (2016). Investigating the relatedness of enteroinvasive *Escherichia coli* to other *E. coli* and *Shigella* isolates by using comparative genomics. Infect Immun.

[17] van den Beld MJ, Reubsaet FA (2012). Differentiation between *Shigella*, enteroinvasive *Escherichia coli* (EIEC) and noninvasive *Escherichia coli*. Eur J Clin Microbiol Infect Dis.

[18] WHO (2017). Global priority list of antibiotic-resistant bacteria to guide research, discovery, and development of new antibiotics.

[19] EU. Comission Implementing Decision (EU) 2018/945 of 22 June 2018 on the communicable diseases and related special health issues to be covered by epidemiological surveillance as well as relevant case definitions 2018 [Updated 6 July 2018.

[20] RIVM (2017). LCI Richtlijn shigellose.

[21] CDC (2017). Shigellosis (*Shigella spp.*) 2017 Case Definition.

[22] CDNA (2018). Shigellosis Surveillance Case Definition.

[23] Van Lint P, De Witte E, Ursi JP, Van Herendael B, Van Schaeren J (2016). A screening algorithm for diagnosing bacterial gastroenteritis by real-time PCR in combination with guided culture. Diagn Microbiol Infect Dis.

[24] de Boer RF, Ott A, Kesztyus B, Kooistra-Smid AM (2010). Improved detection of five major gastrointestinal pathogens by use of a molecular screening approach. J Clin Microbiol.

[25] Silva RM, Toledo MR, Trabulsi LR (1980). Biochemical and cultural characteristics of invasive *Escherichia coli*. J Clin Microbiol.

[26] Escher M, Scavia G, Morabito S, Tozzoli R, Maugliani A, Cantoni S (2014). A severe foodborne outbreak of diarrhoea linked to a canteen in Italy caused by enteroinvasive *Escherichia coli*, an uncommon agent. Epidemiol Infect.

[27] Herzig CTA, Fleischauer AT, Lackey B, Lee N, Lawson T, Moore ZS (2019). Notes from the field: Enteroinvasive *Escherichia coli* outbreak associated with a potluck party - North Carolina, June-July 2018. MMWR Morb Mortal Wkly Rep.

[28] DuPont HL, Formal SB, Hornick RB, Snyder MJ, Libonati JP, Sheahan DG (1971). Pathogenesis of *Escherichia coli* diarrhea. N Engl J Med.

[29] Newitt S, MacGregor V, Robbins V, Bayliss L, Chattaway MA, Dallman T (2016). Two linked Enteroinvasive *Escherichia coli* outbreaks, Nottingham, UK, June 2014. Emerg Infect Dis.

[30] Platts-Mills JA, Babji S, Bodhidatta L, Gratz J, Haque R, Havt A (2015). Pathogen-specific burdens of community diarrhoea in developing countries: a multisite birth cohort study (MAL-ED). Lancet Glob Health.

[31] Tai AY, Easton M, Encena J, Rotty J, Valcanis M, Howden BP (2016). A review of the public health management of shigellosis in Australia in the era of culture-independent diagnostic testing. Aust N Z J Public Health.

[32] Lede IOK-DM, van den Kerkhof JHTC, Notermans DW (2012). Gebrek aan uniformiteit bij meldingen van Shigatoxineproducerende *Escherichia coli* en *Shigella* aan en door GGDen. Infect Bull.

[33] Quinn E, Najjar Z, Huhtinen E, Jegasothy E, Gupta L (2019). Culture-positive shigellosis cases are epidemiologically different to culture-negative/PCR-positive cases. Aust N Z J Public Health.

[34] van den Beld MJC, de Boer RF, Reubsaet FAG, Rossen JWA, Zhou K, Kuiling S (2018). Evaluation of a culture dependent algorithm and a molecular algorithm for identification of *Shigella spp*., *Escherichia coli,* and enteroinvasive *E. coli* (EIEC). J Clin Microbiol.

[35] Haagsma JA, Geenen PL, Ethelberg S, Fetsch A, Hansdotter F, Jansen A (2013). Community incidence of pathogen-specific gastroenteritis: reconstructing the surveillance pyramid for seven pathogens in seven European Union member states. Epidemiol Infect.

[36] de Wit MA, Kortbeek LM, Koopmans MP, de Jager CJ, Wannet WJ, Bartelds AI (2001). A comparison of gastroenteritis in a general practice-based study and a community-based study. Epidemiol Infect.

[37] Freedman SB, Eltorky M, Gorelick M (2010). Pediatric emergency research Canada gastroenteritis study G. evaluation of a gastroenteritis severity score for use in outpatient settings. Pediatrics.

[38] Bruijnesteijn van Coppenraet LE, Dullaert-de Boer M, Ruijs GJ, van der Reijden WA, van der Zanden AG, Weel JF (2015). Case-control comparison of bacterial and protozoan microorganisms associated with gastroenteritis: application of molecular detection. Clin Microbiol Infect.

[39] R_core_team (2018). R: a language and environment for statistical computing. R Foundation for Statistical Computing.

[40] Brenner DJ (1978). Characterization and clinical identification of *Enterobacteriaceae* by DNA hybridization. Prog Clin Pathol.

[41] FaS S, Garrity GM (2005). *Genus* I. Escherichia Castellani and Chalmers. Bergey’s Manual of Systematic Bacteriology. 2 The *Proteobacteria*.

[42] Khan WA, Griffiths JK, Bennish ML (2013). Gastrointestinal and extra-intestinal manifestations of childhood shigellosis in a region where all four species of *Shigella* are endemic. PLoS One.

[43] Liu J, Platts-Mills JA, Juma J, Kabir F, Nkeze J, Okoi C (2016). Use of quantitative molecular diagnostic methods to identify causes of diarrhoea in children: a reanalysis of the GEMS case-control study. Lancet..

[44] Lindsay B, Saha D, Sanogo D, Das SK, Omore R, Farag TH (2015). Association between Shigella infection and diarrhea varies based on location and age of children. Am J Trop Med Hyg.

[45] Liu J, Almeida M, Kabir F, Shakoor S, Qureshi S, Zaidi A (2018). Direct Detection of Shigella in Stool Specimens by Use of a Metagenomic Approach. J Clin Microbiol.

